# Commentary: Three questions for the study of traumatic brain injury in animals

**DOI:** 10.1002/ar.25465

**Published:** 2024-05-23

**Authors:** Gregory Hollin

**Affiliations:** ^1^ Department of Sociological Studies University of Sheffield Sheffield UK

It will likely not be news to this audience that, for much of the 21st century, sports have been in the midst of a “concussion crisis” (e.g., Carroll & Rosner, 2012; Malcolm, 2020; Nowinski, 2007). This crisis has a number of constituent parts, including an increasing concern with the acute effects of brain injury. Nonetheless, the links between brain trauma and neurodegenerative disease—most prominently an Alzheimer's‐like dementia known as Chronic Traumatic Encephalopathy, or CTE—holds center‐stage.

Indeed, a 2020 article in the *Washington Post* described the link between dementia and concussion as:

the most important sports story of the 21st century… [C]oncerns about CTE have inspired a global revolution in concussion safety and fueled an ongoing existential crisis for American's most popular sport. (Hobson, 2020)

And while, as this quote suggests, attention has often centered upon American football, concussion is presented in popular media as offering a threat across a multitude of sports, including Australian rules football (Belson, 2019), bobsled (Futterman, 2020), boxing (Dixon, 2021), bull riding (King, 2024), cricket (Forsaith, 2019), cycling (Warwick, 2021), equestrianism (Finley, 2023), ice hockey (Searing, 2023), mixed martial arts (Gross, 2020), motor sport (PA News Agency, 2022), Olympic diving (Wharton, 2021), professional wrestling (Collins, 2021), rugby league (Pengilly, 2019), rugby union (Peters, 2023), skeleton (Futterman, 2020), soccer (Gill, 2022), sumo wrestling (McCurry, 2021), surfing (Australian Associated Press, 2023), synchronized swimming (Belson, 2016), volleyball (Hruby, 2020), and, I'm sure, many others.

In fact, in the 2020s, it feels increasingly inappropriate to talk about a concussion crisis *in sport*. In 2021, CTE was posthumously diagnosed in a victim of domestic abuse (Danielsen et al., 2021) and the risk of brain injury resulting from intimate partner violence is increasingly being foregrounded (For historical and social scientific work on the relationship between gender and brain injury see, for example: Casper & O'Donnell, 2020; Henne, 2020). Furthermore, and as I write, there is a renewed focus upon the effects of brain injury suffered as part of military activity—a focus which follows suggestions that an army reservist who killed 18 people in a mass shooting may have been exposed to as many as 10,000 blasts on a grenade training range (Philipps, 2023).

Given this cultural milieu, it is unsurprising to see a vast, interdisciplinary body of science developing that aims to better understand the links between brain trauma and neurodegenerative disease. It is similarly unsurprising that some of these scientists aim to achieve insight through the use of animal modeling. I am a sociologist of science, and my research involves observing laboratory work and speaking to scientists about their own research. For several years I have worked with scientists exploring the relationship between traumatic brain injury and neurodegenerative disease, including a number who engage in various forms of animal modeling.

There is nothing new in the idea that we can study both neurodegenerative disease and traumatic brain injury through animal modeling. In the case of the former we need only think of the various mouse models of Alzheimer's Disease; in the case of the latter we can go back to studies on automotive safety that have long involved pigs, baboons, and various other species (e.g., Mertz et al., 1982). (Or simply peruse the abstracts for the latest Society for Neuroscience conference.) Nonetheless, when one speaks to animal modelers or goes into their laboratories, there is a distinct sense that when it comes to animal modeling and the long‐term effects of brain injury, we are dealing with a *nascent* research area in which comparatively little is known and a great deal remains uncertain. In the rest of this commentary, I take these claims of newness and uncertainty seriously, and (re)pose three questions which, based upon my work, researchers ask most frequently and, in turn, seek to answer. My hope is that, first, scientists will recognize these questions from their own lab' discussions and, second, that posing these questions in a clear and public forum will contribute to a healthy debate about the nature of research in this area.

## 1 QUESTION 1: WHAT MAKES A GOOD MODEL OF CTE AND WHY?

One obvious concern within the field is that there may be divergent views, and significant uncertainty, over what constitutes a “good” animal model of CTE. For many researchers, the species of choice are those most commonly used within laboratory settings, most notably mice and rats. There are a host of both pragmatic and epistemological reasons why these species appeal. Pragmatically, they are small, relatively cheap, and have well established care guidelines. Epistemologically, the species are well known both biologically and socially, so an exploration of experimentally‐induced change is easier to conduct. The widespread availability of transgenetic animals provides another level of experimental control (Ojo et al., 2013). These species are examples of what Hans‐Jörg Rheinberger calls “technical objects” (Rheinberger, 1997, p. 29)—they are sufficiently well known that they act as an instrument of knowledge, allowing the effects of intervention to present themselves relatively clearly.

For other researchers, however, no amount of certainty makes up for the fact that many frequently used animal models are profoundly flawed. Chronic traumatic encephalopathy, for example, is definitionally defined by tau depositions at the depth of the sulci (Bieniek et al., 2021). Given that mice and rats are smooth brained, however, we appear to have a fairly fundamental problem. For some of the researchers to whom I've spoken this is a fairly minor matter: rats and mice don't enjoy the taste of alcohol, either, and yet they're frequently used to model alcoholism (Nelson, 2018, p. 145). Primates don't get Parkinson's Disease, and yet they are still used to model it (Giraud, 2019, p. 108). For other researchers, the neuroanatomy of these murine species makes them next to useless.

Scholars who, for one reason or another, reject the use of these standardized animal models frequently turn to the use of novel or unusual species in their work (Ackermans et al., 2021): we see a number of these species drawn upon in this special collection, in fact. When non‐standardized species are utilized, a whole different set of issues emerge. Some researchers, for example, use ferrets: these are gyrencephalitic animals, they're small, and these researchers describe them as an obvious improvement over mice and rats from a neuroanatomical point of view, and over sheep and pigs from an animal husbandry point of view. But these same researchers also describe a great deal of uncertainty about whether the behavioral tests designed for mice and rats—certain mazes, for example—are in any way appropriate with ferrets. How do we know if a ferret is “anxious”? Do ferrets exhibit anxiety in the same way as a rat? Probably not. There may be, then, a need to reimagine a whole suite of behavioral tests.

Other scholars turn to more naturalistic models. An increasing amount of scientific and popular attention has turned towards woodpeckers as an animal model for CTE, for example (Hollin, 2022). Other than the fairly obvious fact that woodpeckers hit their heads a lot, it is still unclear what exactly it is that woodpeckers are taken to model. Is a woodpecker interesting because it has evolved a number of protective mechanisms and therefore doesn't develop any CTE‐adjacent neurodegenerative disease, or is it interesting for precisely the opposite reason, because it *does* suffer brain injury and therefore offers the opportunity to study the long‐term effects of trauma acquired naturally and, perhaps, in a less ethically fraught manner? The woodpecker has, returning to Rheinberger, an “irreducible vagueness” that embodies “what one does not know” (Rheinberger, 1997, p. 28) and this means that a whole lot of work will need to go into describing woodpeckers (and/or ferrets) before we are able to use them to study CTE. And for critics, it will simply never be possible to study the number of woodpeckers necessary to obtain the levels of statistical power needed to answer core questions (or match the insights arising from standardized species).

Historians and philosophers of science have long debated the superior approach to selecting model organisms. Some stress the need for a species to be extensively described prior to transformative insight (Ankeny, 2001). Others stress the value of pluralism and cross‐fertilization (Longino, 2013). Resolving these differences may never be possible, but dialogue and a sense of where others are coming from remains hugely important.

## 2 QUESTION 2: WHAT ARE WE EVEN MODELING?

A second question concerns what, exactly, animals are taken to model. It does not escape the notice of scientists in this area that, approximately once every 15 minutes, there is a new consensus conference intended to bring people together so that we might agree what it is that we're all talking about. We have, for example, the Concussion In Sport Group's quadrennial consensus conference—the most recent of which occurred in 2022 (Patricios et al., 2023)—in which panelists seek to clarify, amongst other things, the definition of concussion and the long term effects of brain trauma. We have the National Institute of Neurological Disorders and Stroke (NINDS)/National Institute of Biomedical Imaging and Bioengineering (NIBIB) consensus statement into the neuropathological criteria for CTE, the second iteration of which was published in 2021 (Bieniek et al., 2021). We have the NINDS consensus criteria for traumatic encephalopathy syndrome, or TES, which is the clinical manifestation of CTE (Katz et al., 2021). I have no doubt that there are many others.

These consensus statements often differ considerably from each other, and there is an increasing suspicion that definitions may be becoming siloed within disciplines. Dominic Malcolm, for example, has observed an increasing tendency on the part of the Concussion In Sport Group to distinguish between “sports‐related concussions” (often abbreviated to “SRCs”) on the one hand, and concussions that result from other forms of activity, on the other. Malcolm (2020, p. 39) suggests that this demarcation may need to be understood as a response to an increasingly fractious relationship between sports scientists and neuroscientists, with the former coining the term “SRC” in order to assert a domain of expertise. It is perhaps unsurprising, therefore, that Concussion In Sport Group consensus statements have, first, been critiqued for failing to represent a consensus and, second, that these critiques are often shouted from across a disciplinary divide. Neuropathologist Willie Stewart, a co‐author on the NINDS/NIBIB definition of CTE, has, for example, been openly critical of the Concussion in Sport Group (Belson, 2022) while other prominent researchers have also elected to ignore their consensus conferences (Bull, 2022). At the same time, those who have not been invited to contribute to the NINDS/NIBIB definition, notably Bennet Omalu, have been critical of that process and the resulting definitions (Hammers & Omalu, 2021; Omalu, 2020). I have also spoken to some researchers who feel incredibly confident that they are able to clinically diagnosis traumatic encephalopathy syndrome and are happy to say that a patient has “probable CTE”; I speak to others who say that this is essentially impossible; that the diagnostic criteria for traumatic encephalopathy syndrome lack sensitivity and specificity; and that we really know next to nothing about the clinical manifestations of CTE.

In many ways, these debates happen at quite some distance from the animal modeling community: consensus committees tend, after all, to exclude all research on non‐human animals when coming to their definitions. Nonetheless, there is a pervasive sense of a moving and diffuse target here. It is clearly important to know what one is modeling, and a sense of working upon shifting sands, necessarily, makes animal research in this area all the harder.

These are not problems unique to the study of brain injury. The writing of contentious consensus making procedures such as the Diagnostic and Statistical Manual for Mental Disorders and the Intergovenmental Panel on Climate Change have been extensively considered (see, for example, Adler & Hirsch Hadorn, 2014 for a consideration of the IPCC; and Pickersgill, 2012, 2024 for a consideration of the DSM). There is not necessarily a need to reinvent the wheel here, but there is, perhaps, a need to get under the hood in order to understand the type of machine we're building.

## 3 QUESTION 3: WHAT CONSTITUTES AN ETHICAL RESEARCH STUDY?

A final area that requires careful consideration concerns the constitution of ethical conduct in relation to the animal modeling of neurodegenerative disease. Rather than re‐litigate any overarching questions about the ethics of animal experimentation, I here want to focus upon two very particular, if quite different, issues that face researchers in this area.

First, while there has been, understandably, a good deal of attention paid to how research is funded (e.g., Bachynski & Goldberg, 2018), broader questions about how scholarship is positioned in relation to sport remains open to question. Many scientists, of course, have a personal relationship with sport. Many researchers play sport themselves, or parent children who do. An even greater number are fans of one sport or another. Furthermore, much research happens amidst sport‐obsessed communities, whether that be soccer in the United Kingdom, football in the United States, or rugby in Australasia. Particularly for academics working in North America, it may also be the case that their home institutions are deeply invested in collegiate athletics.

A number of scholars have argued that these forms of engagement with sport, indeed *any* engagement with sport, is ethically problematic. In an opinion piece for *Scientific American*, for example, Jennifer Tsai and Michelle Morse state that when “we watch and cheer [the NFL], we willfully decide to ignore suffering” and subsequently conclude that the failure of medics and researchers to speak out against collision sport is “a form of sponsorship” bound up with a history of medical “racism and exploitation” (Tsai & Morse, 2020). At the same time, I have been very struck by the number of scientists who, unprompted and shortly after we have met, have gone out of their way to tell me that they do not want to ban sports. I have taken this oft‐offered declaration to mean that at least some researchers see an ethics in *not* taking an overtly antagonistic position when it comes to sport, to recognizing, in some way or another, that research takes place in, and should take account of, communities who enjoy sport. I do not have an answer to this issue (and have numerous unresolved conflicts about my own relationship with sport) but continue to think that an explicit and open discussion is needed.

Second, there is a general sense that deliberately giving animals brain damage, particularly via blunt force trauma, is highly troubling. The devices necessary for the delivery of brain trauma, which often feature animals strapped down and held onto a bed reminiscent, I sometimes think, of a dentist's chair, are undeniably startling. For me, at least, they recall the apparatuses of Harry Harlow and his work that, primatologist Alison Jolly argued, operated at “the limits of ethically permissible animal experimentation” (cited in: Haraway, 1992, p. 409).

I understand, and have a degree of sympathy with, the argument that what I'm describing is an ethics based on appearance. That things look bad, but they're not as bad as they look. That, rationally speaking, it's hard to see how this work is more troubling that any number of other experiments—where animals are infected with diseases, perhaps, or bred to get cancer—that differ mainly in that they are less visceral.

But appearances do matter, and I've noted that neuroscientists, too, seem to be acutely aware of the ethics of their investigation. Conference presentations have proven to be a particular site of interest for seeing how these ethical debates play out in discussions between neuroscientists. Indeed, and on several occasions, I have seen a slightly unusual scene in these settings. First, the speaker will detail their experimental apparatus and procedure. This detailing will often be followed by looks of apparent horror from colleagues in the audience who are unfamiliar with experiments of this type. Possibly pre‐empting any questions that these colleagues might have, the speaker will then clarify that the animals had been rendered unconscious prior to the experiment. In response to *this* revelation, another (usually more senior) colleague will bemoan the fact that the animal was unconscious, arguing that the psychological consequences of experiencing blunt‐force trauma is an important thing to be modeled. The speaker will then absolve themselves of this decision by blaming it entirely on some university ethical review board who mandated that the animals needed to be anesthetized. The speaker, thus, sidesteps an ethical argument by demonstrating an awareness of the controversial nature of this research and their own ethical sensitivity, before sidestepping an epistemological argument by blaming an ethics board.

Again, I have a lot of sympathy with researchers in this situation. On the one hand, all available evidence suggests that the overwhelming majority of those involved in animal experimentation, and who spend significant amounts of time in close proximity to the animals, care deeply about those animals (e.g., Greenhough & Roe, 2011). One the other, one is also expected to recognize that there are scientific norms about a relentless search for the truth. Nonetheless, I do not find this to be a particularly satisfying place to end. It is increasingly recognized that questions of ethics are intimately entangled with questions of epistemics: how one knows is directly related to what one knows (Barad, 2007). An explicit conversation about what ethical animal experimentation should look like, in the context of traumatic brain injury and neurodegenerative disease in particular, continues to be a foundational question in need of further discussion.

In this commentary, I have posed three questions—“What makes a good model of CTE and why?”; “What are we modelling?”, and “What constitutes ethical research?”—that I have heard frequently in my discussions with researchers studying traumatic brain injury and neurodegenerative disease. My hope is that readers will see the relevance of these questions when reading the rest of this special issue and, perhaps, recognize them from their own work. As this special issue shows, this is still a nascent field of research and there is thus the opportunity to imagine things differently, to make clear‐eyed decisions about what the future of the field will look like. Having hard discussions about difficult questions will, I think, be key to this work.

## AUTHOR CONTRIBUTIONS

**Gregory Hollin:** Conceptualization; data curation; formal analysis; funding acquisition; investigation; methodology; project administration; resources; writing – original draft; writing – review and editing.
